# Retraction: Matrix Metalloproteinase-3 in Odontoblastic Cells Derived from Ips Cells: Unique Proliferation Response as Odontoblastic Cells Derived from ES Cells

**DOI:** 10.1371/journal.pone.0198542

**Published:** 2018-06-01

**Authors:** The PLOS ONE Editors

Following publication of this article [1], the authors requested its retraction due to concerns raised about several figures. Aichi Gakuin University investigated this matter and confirmed the following issues.

1. In several cases, the grayscale of a PCR gel image was inverted, and the consequent image was used to represent Western blot data.
  - In Figure 1B, the β-tubulin Western blot panels for Odontoblasts derived from iPS cells and from B6G-2 cells were generated from the GAPDH PCR image shown in Figure 1A for the B6G-2 experiment.
  - In Figure 2, the gray scale of each PCR image in panel A was reversed; the resultant images were adjusted for contrast and black level and were used to represent western blot images in panel B.
  - The gray scale of the GAPDH panel for KN-3 cells in Figure 6A was reversed, and the resultant image was used to generate the β-tubulin panel for the E14Tg2a cell experiment in Figure 6B.
  - In Figure 6, the gray scale of lanes 1–4 of the MMP-3/KN-3 PCR image in Figure 6A was reversed. The resultant image was adjusted for contrast and black level, vertically enlarged, and used as lanes 1–4 of the MMP-3/E14Tg2a blot in Figure 6B.
2. In Figure 6A, vertical lines suggestive of image splicing were observed in the 1st, 4th, 5th, and 7th panels (MMP-3/iPS, GAPDH/B6G-2, MMP-3/E14Tg2a, and MMP-3/KN-3, respectively).
3. In Figure 6A, the same image was used in the 2nd and 6th panels (GAPDH/iPS, GAPDH/E14Tg2a), with the 6th panel rotated 180 degrees relative to the 2nd panel.
4. In Figure 6A, the first four lanes in the 1st and 7th panels (MMP-3/iPS, MMP-3/KN-3) appear to present duplicate data, with the image adjusted for contrast and brightness and vertically enlarged in the 1st panel relative to the 7th.

The original data underlying these results are not available.

In light of these concerns, and in line with the University’s recommendation, the *PLOS ONE* Editors retract this article.

NO, MM, HY, RK, KN, AK, HN agreed with the retraction. TH did not respond.
